# An Update on the Roles of Non-*albicans* *Candida* Species in Vulvovaginitis

**DOI:** 10.3390/jof4040121

**Published:** 2018-10-31

**Authors:** Olufunmilola Makanjuola, Felix Bongomin, Samuel A. Fayemiwo

**Affiliations:** 1 Department of Medical Microbiology and Parasitology, University of Ibadan, Ibadan 200284, Nigeria; dayteet@yahoo.com; 2 Department of Medical Microbiology and Immunology, Gulu University, Gulu P.O. Box 166, Uganda; felix.ayoli9@gmail.com; 3 Faculty of Biology, Medicine and Health, University of Manchester, Manchester M13 9PL, UK

**Keywords:** vulvovaginal candidiasis, *Candida glabrata*, genital infection, vaginal candidosis, antifungal treatment

## Abstract

*Candida* species are one of the commonest causes of vaginitis in healthy women of reproductive age. Vulvovaginal candidiasis (VVC) is characterized by vulvovaginal itching, redness and discharge. *Candida albicans*, which is a common genito-urinary tract commensal, has been the prominent species and remains the most common fungal agent isolated from clinical samples of patients diagnosed with VVC. In recent times, however, there has been a notable shift in the etiology of candidiasis with non-*albicans Candida* (NAC) species gaining prominence. The NAC species now account for approximately 10% to as high as 45% of VVC cases in some studies. This is associated with treatment challenges and a slightly different clinical picture. NAC species vaginitis is milder in presentation, often occur in patients with underlying chronic medical conditions and symptoms tend to be more recurrent or chronic compared with *C. albicans* vaginitis. *C. glabrata* is the most common cause of NAC-VVC. *C. tropicalis*, *C. krusei*, *C. parapsilosis*, and *C. guilliermondii* are the other commonly implicated species. Treatment failure is common in NAC-VVC, since some of these species are intrinsically resistant or show low susceptibilities to commonly used antifungal agents. This article reviews the etiology, pathogenesis, clinical features, diagnosis, and management of NAC vulvovaginitis.

## 1. Introduction

Vulvovaginal candidiasis (VVC) also known as vulvovaginal candidosis or *Candida* vaginitis is a common clinical condition with symptoms and signs of vaginal and vulval inflammation in the presence of *Candida* species [1].

At least one episode of VVC is experienced in up to 70% to 75% of women in the reproductive age group in their lifetime [2]. Epidemiological information suggests that half of all women would have experienced at least one episode of VVC by the age of 25 years with the onset of sexual activity being an important risk factor in this group of individuals [3].

Those who have had at least four episodes of VVC during a 12-month period are considered to have recurrent VVC (RVVC). Up to 10% of women of reproductive age group have this infection as a recurrent condition, and it therefore translates to about 140 million of women being affected worldwide [4,5,6]. Depending on the location, the *Candida* species is now ranked as either primary cause or the second most common cause of vaginal infections accounting for 20–30% of these infections [7,8,9]. Traditionally, *C. albicans* has been regarded as the principal etiologic agent of VVC; however, non-*albicans Candida* (NAC) species have recently gained scientific and epidemiological interests as their prevalence is on the increase globally. NAC species are identified more often in complicated VVC than uncomplicated cases [10]. Most cases of NAC vaginitis are due to *C. glabrata*, *C. krusei*, *C. parapsilosis*, *C. tropicalis*, *C. dubliniensis*, with *C. glabrata* predominating [7,11]. Clinical picture of NAC vaginitis is quite varied, it tends to be milder in severity than *C. albicans* infection but may also be indistinguishable. Diabetes mellitus, older age, prior antifungal agents use, and low socioeconomic status are conditions that result in higher likelihood of non-*albicans* candidiasis. Pathogenic mechanisms of non-*albicans Candida* are not as well understood as those of *Candida albicans* where more extensive research has been carried out. Adhesion, epithelial invasion, and secretion of enzymes play important roles in pathogenicity. *C. glabrata* uses immune evasion strategy as a pathogenic mechanism. An important characteristic of NAC species is intrinsic resistance or low dose susceptibility to the azole antifungals, the first-line treatment, resulting in treatment failure [12]. Identification and antifungal susceptibility testing are necessary for optimal treatment of these infections, especially in settings where diagnosis is based on clinical presentation or limited laboratory testing.

## 2. Classification of Vulvovaginal Candidiasis

An internationally acceptable classification of VVC was adopted over a decade ago [1]. VVC is classified as either uncomplicated or complicated disease based on clinical presentation, host factors, microbiology, and response to therapy [1,13].

Most women with VVC suffer from uncomplicated vaginitis. This presents as sporadic cases of mild to moderate infections usually due to *C. albicans*, and these cases occur predominantly in healthy adult women who have no predisposing factors. On the other hand, about 10–20% of women suffer from complicated VVC that is characterized by more severe attacks or are caused by NAC species with diagnostic and therapeutic implications [10,14]. Infections in pregnancy or associated with or other concurrent conditions, such as immunosuppression and uncontrolled diabetes, are also categorized as complicated infections [1].

## 3. Transmission

*Candida* species colonize the genital tract of women and studies have indicated that the organism may be isolated from the genital tract in 10–55% of healthy, asymptomatic women of child-bearing age where it may result in symptomatic infection when the balance between *Candida* and the vaginal defense factors is altered [7,15,16]. *Candida* species also colonize extragenital sites such as such as the oral cavity and the rectum where it exists as indigenous microbiota of the gastrointestinal tract. *Candida* may also be found as colonizers of the perianal skin and lower urinary tract. Vertical transmission of *Candida* from mother to infant during childbirth birth allows early establishment of colonization of these various sites where its interaction with other resident microbiota and host immunity prevent transition from commensal organisms to pathogens [17]. These sites of colonization have also been implicated as reservoir of genital recolonization in patients with RVVC [18].

Although *Candida* species are not regarded as classical sexually transmitted pathogens, there is some evidence that this organism can be transmitted by both oral and insertive vaginal intercourse [15,19,20,21].

## 4. Etiology of Non-albicans Candida Vaginitis

Most cases of VVC are still caused by *C. albicans*, however, the NAC species are increasingly being implicated in the etiology of VVC [11,12,22,23,24].

Several NAC species have been identified in VVC with the most common including *C. glabrata*, *C. krusei*, *C. parapsilosis*, *C. tropicalis*, *C. dubliniensis*, *C. africana*, *C. duobushaemulonii*, and *C. auris* [7,11,25,26,27]. *C. glabrata* is the most prevalent cause of NAC-vaginitis [28]. As a result of an earlier observation that it lacked pseudohyphae formation, *C. glabrata* was initially classified in the genus *Torulopsis*, which was described in 1894. The genus *Candida* was named much later in 1913 and recent studies have shown that the organism capable of pseudohyphae and even germ tube formation [10,29]. *C. krusei* has been reported as an uncommon cause of vaginitis [8,30,31]. It a cause of refractory vaginitis due to intrinsic azole resistance, hence poor response to conventional antimycotic agents and is commoner in older white women [32]. *C. parapsilosis* is also isolated in *Candida* vaginitis on vaginal fungal culture although this is a relatively infrequent occurrence [13]. However, it was identified as the most frequent *Candida* species in one study conducted at a tertiary care center in North India [23]. *C. parapsilosis*, which was earlier separated into three groups has now been divided into three distinct, closely related species, *C. parapsilosis*, *C. orthopsilosis*, and *C. metapsilosis* [33]. *C. dubliniensis* is similar to *C. albicans* phenotypically, resulting in the underreporting of this organism on routine diagnosis in the laboratory [34]. It is noted to be quite common in HIV-positive individuals [35]. *C. tropicalis* is a NAC species with the highest similarity to *C. albicans* genetically [36]. *C. africana*, produces germ tubes like *C. albicans* but it does not produce chlamydospores. It was first isolated from patients in Africa and Germany, has been reported to cause of VVC in patients in these countries and also in Spain and Italy [37,38]. *C. duobushaemulonii* and other members of the *Candida haemulonii* species complex rarely cause infections in humans. Infection is however more likely in patients with diabetes mellitus [39]. *C. auris* is increasingly being reported from a variety of clinical samples as a cause of candidiasis with varying degrees of severity [40]. It is better known as a causative agent of candidaemia but has also been reported as a cause of VVC [41,42]. Less common species identified as causes of NAC vaginitis include *C. guilliermondii*, *C. famata*, *C. lusitaniae*, *C. norvegiensis*, *C. nivariensis* and *C. bracarensis*, and *C. inconspicua* [27,28,29,30,31,32,33,34,35,36,37,38,39,40,41,42,43].

## 5. Clinical Presentation

Clinical presentation varies from sporadic occasional episodes of mild infection to severe and frequent symptoms [13]. Vaginal *Candida* infection usually presents as vaginitis with intense vulval itching/irritation often accompanied by curdy-white scanty to copious discharge [44,45].

NAC infections generally present with milder symptoms when compared with *C. albicans* vaginitis [13,46]. This is in keeping with findings that NAC species are more frequently isolated from asymptomatic women than those with symptomatic infections [22]. Superficial dyspareunia, pain at the vaginal opening during intercourse, is also commoner in *C. albicans* vaginitis than that caused by NAC species [5]. Patients with underlying medical conditions are more likely to have NAC species infections [5]. Chronic infections are also more likely to have NAC species than acute infections as a significant proportion of these infections result from NAC species, even though *C. albicans* still predominates [47].

In spite of these general features, several reports have found contrasting pictures. Grigoriou et al. [48] reported that more frequent vaginal soreness and painful intercourse were caused by NAC than by *C. albicans*. In that study, *C. glabrata* and *C. krusei* were the predominant NAC species identified. Some researchers have also noted the clinical syndromes were either indistinguishable between those caused by NAC species and those by *C. albicans* or had very subtle differences [5,47]. Clinical features therefore appear unreliable in differentiating *C. albicans* from NAC infections and it is therefore recommended that the diagnosis of infection should not be on the basis of clinical features alone.

Recurrent vulvovaginal candidosis (RVVC) is defined as the occurrence at least four episodes of VVC in a one-year-period [1]. Recurrent infection as seen in RVVC, unlike persistent infection, is characterized by the presence of intervals when the patient is free of symptoms [49]. Recurrent infections have a mutual relationship with NAC species infection where the occurrence of one predisposes to the other. Recurrent infections commonly lead to increased frequency of NAC species colonization, and also, the persistence of NAC species due to their resistance to antifungal agents might result in recurrent infections. This relationship is more common with *C. glabrata* [15,49]. The prevalence of NAC species usually increases with each subsequent episode of VVC in those with recurrent infections. A study that quantified this rate found at least 10% increase in a population of HIV negative women, which was unusually higher than the finding in HIV positive women [15].

## 6. Epidemiology of Non-albicans Vulvovaginal Candidiasis

Epidemiological data in the last two decades have revealed a striking mycological shift in regards to geographic variation in etiologic species of VVC. There is a rising prevalence of infections caused by NAC species, while that of *C. albicans* is decreasing in spite of retaining its position as the most common *Candida* species in general [44,50]. This trend is not limited to vaginal infections but has been reported in infections in many other sites of the body [11,51,52,53]. As a result of this, other species, predominantly *C. glabrata*, *C. krusei*, *C. parapsilosis*, and *C. tropicalis*, are gaining clinical recognition [52].

This change has been fueled by the widespread and indiscriminate use of broad-spectrum antibiotics coupled with the increased availability of both over-the-counter and prescribed antimycotic agents [54]. An earlier study that was published in 1991 by Horowitz and colleagues reported an increase in the occurrence of VVC due to NAC species from about 10% in the 1970s to up to 20% in the 1980s [55]. A more recent multicenter study in support of this observation noted that during the time periods of 1997–2000 and 2005–2007 the rates of isolation of *C. glabrata*, *C. tropicalis*, and *C. parapsilosis* increased from 10.2% to 11.7%, 5.4% to 8.0%, and 4.8% to 5.6%, respectively [56]. The epidemiological trend is that VVC that is caused by NAC species is associated with higher frequency of recurrence [16,27].

A single general estimate of the global prevalence of NAC species in VVC is difficult to establish. There is limited data on the actual epidemiology and distribution of the various non-*albicans Candida* species in vulvovaginitis. Table 1 is a summary of some studies on the prevalence of the various NAC species in various patient populations. In women with VVC, the distribution of NAC species identified varies widely depending on the populations studied and also the locations and may account for the findings. However, from most published studies, a range of as low as approximately ~10% to, in some studies, as high as 45% have been reported (Table 1). [44]. In contrast to the predominance of *C. albicans*, Deorukhkar and colleagues reported that NAC species accounted for over 60% of their isolates, while Sangare et al found non-*albicans* species predominating in their study population of pregnant women [11,57]. NAC species are more likely to be isolated from samples of patients with multiple positive cultures than from samples with only one organism cultured, a picture that is commoner in the older age group [12].

In general, *C. glabrata* is the most common NAC species, causing vaginitis, accounting for half to two-thirds of cases in majority of reports of NAC vaginitis, [7,12,13,28]. Rarely, other NAC species have been reported as the most prevalent, such as that by Jackson et al. [54] where *C. tropicalis* was their predominant species, followed by *C. glabrata*. Also, a study among pregnant women identified *C. tropicalis* as the most prevalent *Candida* species, higher than *C. albicans* [8]. Another study from Belgium reported *C. guilliermondii* isolates in 17.3% among patients with symptomatic VVC [27].

There is no clear second most common NAC species as different studies have documented *C. tropicalis*, *C. krusei*, and *C. parapsilosis* in varying proportions [7,8,30,31,44]. *C. krusei* is generally of low prevalence [8]. Table 1 is a summary of the epidemiology of non-*albicans* vaginal candidiasis across some locations globally between 2001 and 2018 (up to May).

## 7. Pathogenesis

*Candida* species exists as a normal commensal in many parts of the body, including the female genital tract with its growth being continually controlled and limited by the innate immune system and complex bacterial microbiome dynamics [16].

Many factors have been attributed to the pathogenic potentials of *Candida* species, some of which includes adhesion molecules that aid in the invasion of the organism into the host epithelial cells, secretion of various enzymes e.g., hydrolases, yeast-to-hypha transition, phenotypic switching, biofilm formation, contact tracing, and thigmotropism. The most critical virulent factors in the pathogenesis of VVC include the cell wall, adhesion, and extracellular proteolytic enzymes.

The cell wall of *Candida* species is one of the components useful for the success of the organism as a pathogen. It is composed of polysaccharides, mannan, glucan, and chitin. Mannan (23%) and glucan (40–60%) form the substantial proportion of the cell wall composition. Attachment of the *Candida* cell wall to the vaginal epithelium is the initial step that distinguishes colonisation from infection Immune recognition of *Candida* species is dominated significantly by the biding of the fungal cell wall carbohydrates, which is mediated by fungal pathogen-associated molecular patterns (PAMPs) engagement with the host pattern recognition receptors (PRRs).

Adherence of the *Candida* species to the vaginal epithelial surface also results in the successful colonisation or infection. *C. albicans* adheres significantly in higher numbers than the non-*Candida albicans* species, like *C. glabrata*, *C. parapsilosis*, *C. krusei*, and *C. guilliermondii*. The mechanism of this adherence contributes to the persistence of *Candida* species within the host and this is crucial to the establishment of the colonisation, as well as infection. The specific cell proteins called adhesins mediate these attachments to the epithelial surface. Adhesins usually recognise the host ligand, extracellular matrix, and promote the binding to abiotic surfaces [56].

Most of the enzymes that are expressed by *Candida* species are involved in the degradation of the connective tissues and cleavage of the host immune defense proteins, which in turn, enhance the nutrition acquisition, fungal invasion, and evasion of the host immune system. Of all the hydrolases, aspartyl proteases have demonstrated to have significant roles, which range from inactivation of Factor-H to: complement receptors CR3 and CR4 on macrophages. These activities mediate the escape of *Candida* species from the innate immune system’s recognition.

Most data on *Candida*-epithelial surface interactions have been on research using *Candida albicans* and information is deficient on such interactions in NAC species. Following colonization, various pathogenic mechanisms are involved in interactions of candida with the vaginal epithelium, including infiltration and invasion, induced endocytosis, secretion of proinflammatory cytokines and hydrolytic enzymes, epithelial damage, and reduction in mucosal barrier function. These *Candida*-epithelial interactions vary between different species and even different strains of same species [68].

*C. albicans* ability to switch between yeast and hyphal morphologies is central to its virulence. This yeast- hyphal switch is controlled by environmental sensors and regulatory transcriptional factors. The resultant morphogenic responses contribute to the immunopathology of the vaginitis in VVC through the Efg1 and the Bcr1 pathways expression [69]. Recently, Candidalysin, a peptide toxin secreted during hyphal formation was discovered in *C. albicans* as a virulence factor in this organism. Candidalysin is a crucial in immunopathology of vulvovaginal candidiasis, as it promotes epithelial damage, stimulates immune activation, and phagocyte function [70].

*C. glabrata* is able to survive in macrophages as an immune evasion strategy, thus avoiding the innate immune response to pathogens. This adaptation to intracellular survival is related to its ability to prevent toxic phagolysosome environments by modifying its phagosome, suppressing ROS production and producing minimal proinflammatory response [71].

*C. dubliniensis* genome is very similar to that of *C. albicans* but in terms of pathogenicity, both species differ considerably. One of the reasons that could account for the higher pathogenicity in *C. albicans* is the absence of ALS3, gene, a protein that is involved in epithelial adhesion, in *C. dubliniensis* [68].

More extensive research on virulence has been has been carried out on *C. albicans* as compared to the NAC species and *C. albicans* is able to express all virulence factors. The NAC species exhibit various combinations of virulence factors depending on the strain [72].

Hyphae production though a major factor in *C. albicans* infection plays little if any role in majority of NAC vaginitis [73]. Phospholipase activity has been noted to enhance adherence to host cells and also the production of biofilms. It has been postulated that, during infections, adhesion to host cell membranes and lysis are mediated by phospholipases [74]. Phospholipase production is better in *C. albicans* in comparison to other species of *Candida*; a study showed that 53% of the tested NAC species had detectable phospholipase activity [74,75].

A study conducted to demonstrate the virulence factors in various *Candida* species documented that among the NAC species, all were able to demonstrate some of the tested virulence factors in varying proportions. Biofilm production was noted as highest in *C. tropicalis* but was not seen in *C. dublinensis*, and *C. glabrata* demonstrated the highest hemolysin production. The NAC species generally produced low levels of coagulase production and coagulase was undetectable in *C. kefyr* and *C. dublinensis* [11]. High-frequency reversible phenotypic switching, which is important in *C. albicans*, has also been demonstrated in *C. tropicalis* [76]. Hemolysin production by NAC has been found to be comparable to that of *C. albicans* [75].

## 8. Risk Factors

Risk factors that are commonly associated with VVC include the recent prolonged use of broad-spectrum antibiotics, extreme of ages, poorly controlled diabetes mellitus, oral contraceptive use and pregnancy [48].

Antibiotics use is associated with both an increased prevalence of vaginal colonization by *Candida* and increased incidence of symptomatic infection during the period of antibiotic therapy [77]. Use of azole antimycotics, especially low dose maintenance regimens predispose to NAC vulvovaginitis [8,11]. Many topical azole antifungals are available as over-the-counter medications, which has greatly increased their use, thereby increasing the prevalence of NAC vaginitis [12].

Diabetes mellitus (DM) also results in both increased rate of vaginal *Candida* colonization and infection with *Candida* [11]. Poorly controlled DM causes increased glycogen levels and other metabolic alterations, which lower vaginal P^H^ resulting in *Candida* colonization at a rate higher than that of commensal bacteria (vaginal dysbiosis) and infection [78]. A significant proportion of VVC in diabetic patients is caused by NAC species, especially *C. glabrata* and *C. tropicalis* [79]. Both Type 1 and Type 2 diabetes patients are prone to NAC VVC, but there are conflicting reports as to which group has a higher predisposition to NAC species infection than the other [30,78]. Usage of estrogen also predisposes to candidiasis by stimulating epithelial production of glycogen, thus supplying a rich substrate for *Candida*. Dennerstein and Ellis noted a differential metabolism of glycogen between *C. albicans* and NAC species as all NAC species tested did not metabolize glycogen while *C. albicans* did. This observation may have a bearing on the pathogenicity and proportion of NAC in patients on estrogen therapy.

A relationship exists between VVC and age. Young women aged 21–40 years are significantly more likely than older age groups to suffer from VVC [9,80]. However, older age group has been associated with NAC infections when compared to *C. albicans* [26,46,81]. The practice of vaginal douching has been found to be associated with the development of VVC [11,82] with higher frequency of douching resulting in the higher likelihood of having NAC infection [46,83].

Pregnant women are predisposed to VVC, with a high rate of *Candida* species isolation in both symptomatic and asymptomatic women [84]. A prevalence of VVC of up to 36–37% has been found among pregnant women [85,86]. Infections are more likely in the third trimester than other periods [85]. Although, in general, *C. albicans* is also the most common species among pregnant women, some studies have found NAC species, such as *C. tropicalis*, predominating [8,31,87,88]. Low socioeconomic background, poor education, non-white race, and underling medical conditions are commoner in NAC vaginitis [5,46]. Candidiasis, including VVC, is a common opportunistic infection in HIV positive patients [89]. It has been discovered that, over time, a significant increase in the prevalence of vaginal colonization with NAC species occurs among HIV positive women. This finding is thought to be due to long term administration of fluconazole for prophylaxis against other fungal opportunistic infections in HIV positive individuals [89,90]. A study with comparable findings observed that *C. dubliniensis* infection is less likely in HIV-negative patients than HIV positive [11]. Similarly, low viral load is associated with a low likelihood of vaginal carriage of NAC [89].

## 9. Laboratory Identification

It is necessary to identify the *Candida* species responsible for infections in all patients presenting with VVC especially those with refractory infections. Such identification will influence antimycotic agents selection and duration of therapy [32].

Vaginal and endocervical swab samples are collected and a microscopic examination of these secretions is performed as a wet smear. In some cases, 10–15% potassium hydroxide (KOH) solution is used to disintegrate other cellular elements and thus makes it easier to detect *Candida* yeast cells. A characteristic fishy odor on addition of KOH to the smear, positive “whiff test”, is suggestive of bacterial vaginosis aiding in differentiating the infection from candidiasis. Prepared slides may be examined by light microscopy; however, phase contrast or polarized light microscopy may be employed as these make details easier to observe. Wet mount, which shows only blastospores on microscopy, is usually an early suggestion of NAC species infection; whereas, the presence of hyphae shows that the infection is most likely due to *C. albicans* [32,91]. Smears may also be made, air-dried, and fixed with methanol or heat to be examined by Gram-, Giemsa-, or Papanicolaou staining.

*Candida* species are able to grow well at 25–37 °C on non-selective media, such as blood agar and also Sabouraud’s dextrose agar with or without antibiotics, a medium that is usually used for the selective culture and identification of fungi. Colonies are fast growing with typical yeast colonies appearing within 1–2 days of incubation [92,93].

*Candida* chromogenic media are culture media containing chromogenic substances, with species specific hydrolysis of these substances, resulting in color change. Such media include CHROMAgar *Candida* and ChromID *Candida* agar [94,95]. Chromogenic media are useful for direct speciation of the most common species of Candida on culture and also for the detection of mixed cultures [92,95,96]. Fluconazole may also be added to CHROMAgar for detection of fluconazole resistance in clinical isolates [95,97].

Identification of *Candida* species following its characteristic appearance on media is based on microscopy of the wet mount and Gram stain, which shows budding yeast cells with or without pseudohyphae and hyphae [32,91,93]. Following the morphological identification of *Candida* spp., a germ tube test is carried out and germ tube-positive species are presumptively identified as *C. albicans* [93,98]. However, two NAC species, *C. dubliniensis* and *C. africana*, are also germ tube positive [99,100]. This has often led to the underreporting of these NAC species. *C. dubliniensis* and *C. africana* can be differentiated from *C. albicans* most definitively using molecular testing. Phenotypic tests, such as gas-liquid chromatography, appearance on chromogenic agar, growth on Pal’s, and growth at elevated temperatures can also be used reliably [101,102].

Chlamydospores formation on cornmeal agar also aids in distinguishing NAC from *C. albicans* infection, as *C. albicans* produces chlamydospores on nutrient deficient media [103].

Germ tube-negative colonies are regarded as NAC species and they are further speciated using various identification systems and methods. Several of these commercial identification systems are available such as Rapid ID yeast plus System, API 20 C system, Vitek2 yeast identification system, API ID 32C, Yeast Star, Yeast Plus system, Auxacolor, and API Candida [104,105]. The common *Candida* species are identified equally by the most used tests (Auxacolor, API ID32C and Vitek) while Vitek is said perform best at correctly identifying rare species [105,106].

Molecular biology techniques, such as PCR, are now being used to identify *Candida* species and other sexually transmitted pathogens [107]. Results are variable depending on test sensitivity and specificity [96]. Newer methods, such as real time PCR, restriction fragment length polymorphisms, pulsed-field gel electrophoresis, and randomly amplified polymorphic DNA have faster turnaround times, allow for the identification of a variable number of species and different strains of a specie [10,81,108,109]. It is important not to rely on microscopy, culture, or PCR alone to avoid inaccurate results [96].

## 10. Antifungal Susceptibility Profile of Candida Species

Increasing resistance to antifungal agents especially fluconazole has made antifungal susceptibility testing of *Candida* important. Remarkable progress has been made in antifungal susceptibility testing. Broth dilution, Disk diffusion, microtiter method, and E test are now available with species-specific breakpoints for each agent. Assays for susceptibility to antifungal agents are most carried out for azoles such as fluconazole, itraconazole and/or voriconazole [110].

The Clinical Laboratory Standards Institute (CLSI) and the European Committee on Antibiotic Susceptibility Testing (EUCAST) have standardized methods for antifungal susceptibility testing and developed breakpoints for classifying some *Candida* and NAC species. In *C. albicans*, *C. tropicalis*, and *C. parapsilosis*, the CLSI breakpoints for Fluconazole Susceptible (S), Susceptible Dose Dependent (SDD), and Resistant (R) are ≤2, 4, ≤8 µg/mL, respectively. In *C. glabrata*, MIC values of ≤32 and ≤64 µg/ml are regarded as SDD and resistant, respectively, while *C. krusei* is assumed to be intrinsically resistant to fluconazole, thus MIC interpretations are not recommended [111]. In spite of these standardized methods of testing, interpretation of susceptibility results remain a challenge. NAC species exhibit varying antifungal susceptibility profiles. A significant change in the epidemiologic pattern of *Candida* and also development of resistance among previously susceptible *Candida* species have resulted from an increased availability of both prescribed and over-the-counter antifungal agents [54]. Most NAC species now have higher azole minimum inhibitory concentrations (MICs) thus the resultant infections are usually difficult to treat [12]. Resistance is common in NAC species to the azole group of antifungal agents [11]. A large study found more than two-thirds of their *C. glabrata* vaginal isolates to be fluconazole non-susceptible which was higher than the report for bloodstream infection isolates [12,112]. Even among non-*albicans* vaginal *Candida* isolates with low prevalence of fluconazole resistance, increased dose-dependent resistance has been demonstrated with implications on optimal therapy of vaginitis that is caused by these NAC species [113]. The MICs of some NAC have been noted to increase by several folds following exposure to antifungal agents but this increase may also occur in the absence of such exposure [12]. Organisms from patients with recurrent VVC are more resistant to azoles than those from uncomplicated VVC patients [110].

In *C. glabrata* infections, antifungal susceptibility may be affected by low pH, the usual vaginal environment. In such condition, a progressive increase in MIC for Amphotericin B and azoles are evident when the pH is lowered, though isolates remain susceptible to flucytosine and caspofungin [28]. *C. parapsilosis* isolates have antifungal susceptibility similar to that of *C. albicans* [114]. *C. auris*, an emerging *Candida* specie is reported to be intrinsically resistant to the azoles and amphotericin B [41,42].

## 11. Treatment of Non-albicans Candida Vulvovaginal Vaginitis

First-line treatment for candida vaginitis is with topical antifungal agents, such as clotrimazole, or a single 150-mg oral dose of fluconazole with good response in uncomplicated cases [115]. Unlike *C. albicans* infection where fluconazole is effective in most cases, NAC species infections are not as responsive to fluconazole [12,47,79]. Effective treatment and elimination of NAC, particularly *C. glabrata* from the vagina is difficult, as this first-line treatment is associated with a high failure rate [116,117]. This makes symptomatic NAC vaginitis a significant health problem for the clinician [28]. Alternative treatment options are limited as the current armamentarium of antifungal agents is quite narrow in range.

Nystatin has been found to be an excellent choice for the treatment of NAC vaginitis. One study found that the resistance rate to Nystatin in their isolates was 0.3% among *C. glabrata*, while for *C. krusei* and *C. tropicalis*, it was 3.8% and 0, respectively [50]. Boric acid suppositories are also able to achieve high mycological cure in NAC vaginitis [79]. It is given as 600 mg capsules that are inserted once or twice daily intravaginally for two weeks [12,117,118]. Other treatments that have been found to be effective are 17% flucytosine topically for two weeks, high dose fluconazole (800 mg) daily for 2–3 weeks, Amphotericin B suppositories and oral posaconazole [118,119,120]. Local imidazole, nystatin, ciclopirox olamine, or boric acid should be used for *C. krusei* infection, as it is resistant to the triazoles, such as fluconazole and itraconazole [32,118]. *C. parapsilosis* vaginitis is easily treatable due to its high susceptibility to virtually all antifungal agents [121,122].

Combination therapies of boric acid and amphotericin B have also been tried with excellent results. However, the high cost of this regimen is a major limitation to wide use [117]. Echinocandins, such as micafungin and anidulafungin, are very expensive, though useful for invasive infections are not indicated in vaginal infections due to lack of topical preparations in clinical use [118]. Maintenance therapy or prophylaxis is essential, following treatment of an acute episode of RVVC in both *C. albicans* and NAC infections [49]. Although a cure is difficult to achieve, patients with chronic recurrent VVC may be given weekly or monthly treatment with fluconazole on a long-term basis to reduce the rate of recurrence of symptomatic infection [123]. A regimen of 150mg fluconazole every other day for three doses, which is followed by 150–200 mg fluconazole weekly for six months, is able to prevent further recurrence in over 80% of women [124].

### Supportive Care: Effect of Probiotics

Probiotics are live microorganisms which when they are administered on a host in adequate amounts confer a health benefit [125]. Studies have demonstrated that lactobacilli can inhibit the *Candida* growth and/or adherence to the vaginal epithelium. Probiotics, particularly *Lactobcillus acidophilus*, *L. rhamnosus* GR-1, and *L. fermentum* RC-14, have therefore been considered as potential preventive agents in women who suffering from RVVC [126]. They are administered either orally or intravaginally. Clinical efficacy is however inconclusive as some other studies have not been able to demonstrate the benefit of these probiotics in the prevention of RVVC [127]. Of note is that these trials have been carried out on *C. albicans*, not the NAC species, thus there is lack of information on the effects of probiotics on NAC.

## 12. Conclusions

Vaginal candidiasis is a common infection in women worldwide. *C. albicans* is the most common etiology but there is an ongoing increase in the prevalence of NAC species in vaginal and indeed other candida infections due to increasing use of antifungal agents and susceptible patient population. This trend is unlikely to wane given the circumstances. Large gaps, which need to be filled, exist in our current understanding of the pathogenic mechanisms of non-*albicans Candida* infections Most respond poorly to fluconazole, *C. krusei* is intrinsically resistant to fluconazole, the usual therapy for VVC, resulting in treatment failure and the need for alternative therapy. Definitive laboratory procedures are of paramount importance to identify *Candida* isolates from suspected VVC cases to a species level to ensure appropriate and effective use of antifungal agents.

## Figures and Tables

**Table 1:** Epidemiology of non albicans vaginal candidiasis.

Author (Reference)	Year	Country	Prevalence of Vulvovaginal Candidiasis	Proportion of *C. albicans* (%)	Proportion of Non Albicans Candida Species (%)				
					*C. glabrata*	*C. tropicalis*	*C. krusei*	*C. parapsilosis*	Others
Bitew and Abebaw [58]	2018	Ethiopia	41.4	58.6	3.4	2.3	17.2	2.3	13.9
Jimoh et al. [59]	2016	Nigeria	40.8	48.5	9.2	11.1	-	31.3	-
Alfouzan et al. [60]	2015	Kuwait	13.2	73.9	19.8	0.96	0.96	0.96	1.9
Shi et al. [61]	2015	China	61	91.4	4.3	3.2	-	1.1	-
Kumari et al. [23]	2013	India	30.8	32.4	22.5	-	-	45.1	-
Mahmoudi Rad et al. [62]	2011	Iran	-	67	18.3	6.8	5.8	1.6	0.5
Ahmad et al. [63]	2009	India	20.5	46.9	36.7	2.8	1.4	-	1.9
Ozcan et al. [64]	2006	Turkey	35	75	14	7	3.5	-	-
Pirotta et al. [65]	2006	Australia	21	73	20	-	-	5	-
De vos et al. [27]	2005	Belgium	77.9	78	2.6	-	-	-	18.6
Corsello et al. [66]	2003	Italy	43.5	77.1	14.6	-	4	-	-
Ribeiro et al. [67]	2001	Brazil	60	90	4	4	2	-	-

## Abbreviations

VVC	Vulvovaginal candidiasis
RVVC	Recurrent vulvovaginal candidiasis
NAC	Non-*albicans Candida*
KOH	Potassium hydroxide
PCR	Polymerase chain reaction
DM	Diabetes mellitus
